# Early apoptosis of blood monocytes in the septic host: is it a mechanism of protection in the event of septic shock?

**DOI:** 10.1186/cc4921

**Published:** 2006-05-12

**Authors:** Evangelos J Giamarellos-Bourboulis, Christina Routsi, Diamantis Plachouras, Vassiliki Markaki, Maria Raftogiannis, Dimitrios Zervakis, Vassilios Koussoulas, Stylianos Orfanos, Anastasia Kotanidou, Apostolos Armaganidis, Charis Roussos, Helen Giamarellou

**Affiliations:** 1 4th Department of Internal Medicine, University of Athens, Medical School, Greece; 2 1st Department of Critical Care, University of Athens, Medical School, Greece; 3 2nd Department of Critical Care, University of Athens, Medical School, Greece

## Abstract

**Introduction:**

Based on the central role of the triggering of monocytes for the initiation of the septic cascade, it was investigated whether apoptosis of blood monocytes in septic patients is connected to their final outcome.

**Methods:**

Blood monocytes were isolated from 90 patients with septic syndrome due to ventilator-associated pneumonia on days 1, 3, 5 and 7 from the initiation of symptoms. Apoptosis was defined after incubation with annexin-V-fluorescein isothiocyanate and propidium iodine and reading by a flow cytometer. The function of first-day monocytes was evaluated from the concentrations of tumour necrosis factor alpha (TNFα) and IL-6 in supernatants of cell cultures after triggering with endotoxins. TNFα, IL-6 and IL-8 were estimated in serum by an enzyme immunoassay.

**Results:**

Mortality rates of patients with apoptosis ≤50% compared with patients with apoptosis >50% were 49.12% and 15.15%, respectively (*P *< 0.0001). Kaplan-Meier analysis showed a 28-day survival benefit in patients with septic shock and monocyte apoptosis >50% compared with those patients with apoptosis ≤50% (*P *= 0.0032). Production of IL-6 by monocytes on the first day by patients with apoptosis ≤50% was similar compared with monocytes isolated from healthy controls. Serum concentrations of TNFα were higher in patients with monocyte apoptosis ≤50% and septic shock compared with patients with apoptosis >50% on day 7; similar findings occurred for serum IL-6 on days 1 and 7 and for serum IL-8 on days 1 and 5.

**Conclusion:**

Early apoptosis of monocytes upon presentation of clinical signs of sepsis is connected to a favourable outcome. These findings are of particular importance for the patient with septic shock, where they might constitute a mechanism of pathogenesis.

## Introduction

Apoptotic cascade is a process already described to supervene during the evolution of sepsis in lymphocytes, in tissue macrophages and in intestinal epithelia, and it is connected to organ dysfunction [1]. Although data for the apoptosis of cells of the adaptive immune system are available, little evidence exists for the implication of the innate immune system [2]. The need for knowledge in that field is further aggravated by the central role of monocytes in the pathogenesis of sepsis [3]. The existing theory for the pathogenesis of sepsis is based on the overproduction of proinflammatory cytokines by blood monocytes when triggered by the cell-wall constituents of the bacterial pathogens [4]. The present study aimed to clarify that field with special focus on septic shock. Attention to septic shock was based on the immune imbalance diagnosed early before advent of shock and expressed by a considerable increase of proinflammatory mediators [5].

The main characteristic of the present study was that the entire population enrolled became septic because of the same underlying infection – ventilator-associated pneumonia (VAP). This is a striking difference compared with all other clinical trials on sepsis, and it was based on the need to elaborate an entire study population conferring an antigenic stimulus that did not differ considerably within patients. The enrolment of patients with different types of antigenic stimuli has been implicated as a great disadvantage in the evaluation of clinical trials on sepsis [6].

## Patients and methods

### Study design

A total of 90 patients were enrolled in a prospective study conducted over the period June 2004 to January 2005. Patients were hospitalized in the Department of Critical Care of the 'Evangelismos' General Hospital and in the 2^nd ^Department of Critical Care of the 'ATTIKON' University Hospital of Athens. The study was approved by the Ethics Committee of both hospitals. All enrolled patients were intubated for at least 48 hours prior to enrolment and they were aged older than 18 years. Written informed consent was provided by their first-degree or second-degree relatives in accordance with the Helsinki declaration of 1975. Exclusion criteria were the presence of neutropaenia (<500 neutrophils/mm^3^), HIV infection, and the oral intake of corticosteroids at a dose equal to or higher than 1 mg/kg equivalent prednisone for a period longer than one month.

Inclusion criteria were the concomitant presence of VAP and of sepsis, severe sepsis or septic shock. None of the enrolled patients was suffering from solid tumour malignancy.

Diagnosis of sepsis was based on the presence of at least two of the following [12]: core temperature >38°C or <36°C, _*P*_CO_2 _partial pressure of carbon dioxide <32 mmHg, pulse rate >90/minute, and white blood cells >12,000/μl or <4,000/μl or >10% of bands.

Diagnosis of VAP was established in any patient presenting with the following signs: core temperature >38°C or <36°C, new or persistent consolidation in a lung X-ray, and purulent trancheobronchial secretions (TBS) [7-11].

Severe sepsis was determined as the acute dysfunction of at least one organ. This was evaluated by the acute presentation of at least one of four criteria [12]: acute respiratory distress syndrome, any value of pO_2_/FiO_2 _below 200 with the presence of diffuse shadows in a lung X-ray; acute renal failure, the production of less than 0.5 ml urine/kg body weight/hour for at least 2 hours provided that the negative fluid balance of the patient was corrected; metabolic acidosis, any pH < 7.30 or any base deficit > 5 mEq/l and serum lactate at least more than twice the normal value; and acute coagulopathy, any platelet count <100,000/μl or International Normalized Ratio > 1.5

Septic shock was considered any value of systolic pressure <90 mmHg requiring the administration of vasopressors [12].

Sedation was achieved in all patients with the intravenous administration of midazolame and propofol 1%. Upon enrolment in the study, quantitative TBS cultures were performed. TBS were collected after insertion of a sterile catheter in the intubation tube or in the tracheostomy connected to a negative pressure device. Enrolled patients were followed up on a daily basis for a total of 28 days; evaluation comprised lung X-rays, estimation of the pO_2_/FiO_2 _ratio and of the Acute Physiology and Chronic Health Evaluation II and Sequential Organ Failure Assessment scores. Resolution of VAP was considered as any decrease of X-ray findings accompanied by an increase of the pO_2_/FiO_2 _ratio. Antimicrobial therapy of VAP was selected by attending physicians according to published guidelines [8,9].

Laboratory examinations were performed for seven consecutive days with sampling of 10 ml blood after venipuncure of a peripheral vein under sterile conditions. Isolation of blood monocytes was performed on each second day. Five millilitres of blood were added into commercially available flasks for culture (Becton Dickinson, Cockeysville MD, USA); 3 ml were then collected in a heparinized syringe for isolation of monocytes and 2 ml were collected in a sterile tube. After centrifugation, the serum was kept at -70°C for the estimation of the concentrations of tumour necrosis factor alpha (TNFα), of IL-6 and of IL-8.

### Laboratory techniques

Quantitative TBS cultures were performed after collection. TBS (0.5 ml) was added to a sterile tube with 2 ml dithiothreitol (1 mg/ml; Oxoid Ltd, London, UK) and diluted five consecutive times in the ratio 1:10. Volumes of 0.1 ml each dilution were plated onto McConkey, blood and Saboureaud agar (Becton Dickinson). Dishes were incubated for 5 days at 37°C or 42°C for Saboureaud plates and their count was estimated after multiplying with the appropriate dilution factor. Cultures yielding a pathogen at a count ≥1 × 10^6 ^colony-forming units/ml were considered positive [13]. Flasks with blood were incubated for 7 days. Identification of pathogens was performed by the API20E and the API20NE systems (bioMérieux, Paris, France).

For the isolation of blood monocytes, the collected heparinized venous blood was layered over Ficoll Hypaque (Biochrom, Berlin, Germany) and centrifuged. Isolated mononuclear cells were washed three time with PBS (pH 7.2) (Merck, Darmstadt, Germany) and incubated with RPMI 1640 enriched with 10% foetal bovine serum and 2 mM glutamine in the presence of 100 U/ml penicillin G and 0.1 mg/ml streptomycin (Sigma Co, St Louis, MO, USA) in 25 cm^3 ^flasks. After 1 hour of incubation at 37°C in 5% CO_2_, nonadherent cells were removed; adherent monocytes were thoroughly washed with Hanks' solution (Biochrom). Monocytes were then harvested with a 0.25% thrypsin/0.02% ethylenediamine tetraacetic acid solution (Biochrom) and counted in a Neubauer plate. Their purity was more than 95%, determined after staining with the anti-CD14 monoclonal antibody at the fluorocolour fluorescein isothiocyanate (emission 520 nm; Immunotech, Marseille, France) and reading through the EPICS XL/MSL flow cytometer (Beckman Coulter Co, Miami, FL, USA). Their viability was assessed by trypan blue.

**Figure 1:**
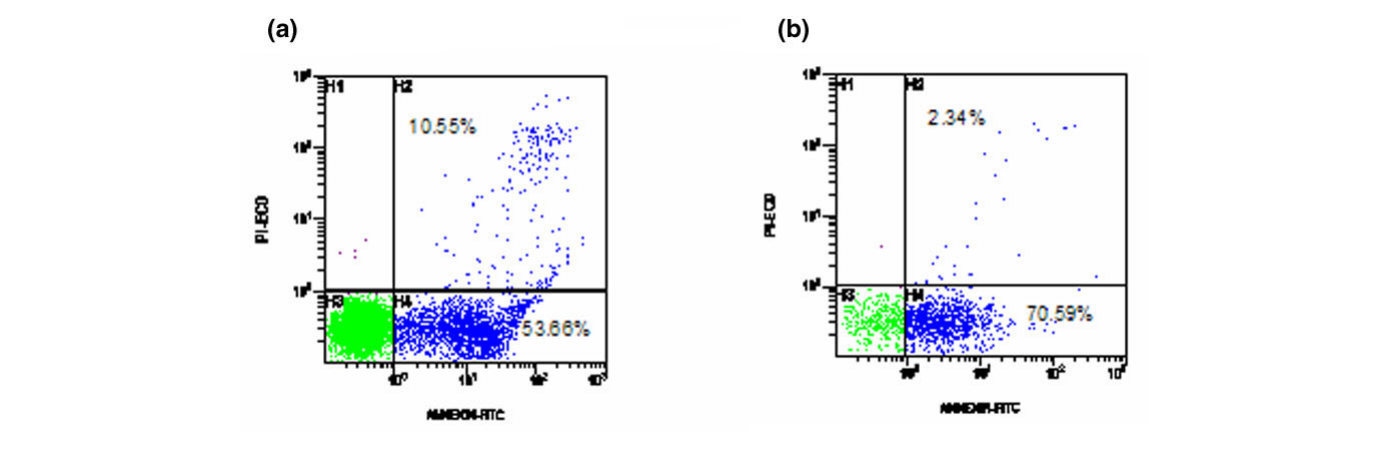
Monocytes apoptosis of two septic patients after flow cytometric analysis; ANNEXIN-V(+)PI(-) cells (region H4) are considered apoptotic.

For the estimation of monocyte apoptosis, cells were incubated for 15 minutes in the dark with the protein annexin-V at the flurocolour fluorescein isothiocyanate (emission 520 nm; Immunotech) and with propidium iodine at the fluorocolour EC5 (emission 550 nm; Immunotech). Annexin-V is connected to the phospatidylserine residue revealed in cell membranes upon initiation of the apoptotic process. Propidium iodine is connected to dead cells. Monocytes staining positive for annexin-V and staining negative for propidium iodine after running through the EPICS XL/MSL flow cytometer (Beckman Coulter Co.) were considered apoptotic (Figure 1). Apoptosis of monocytes was estimated for four healthy volunteers isolated as described earlier.

The function of monocytes was determined only for cells isolated on the first day. After estimation of their apoptosis, monocytes were distributed in two wells of a 12-well plate; they were incubated with RPMI 1640 supplemented with 10% foetal bovine serum and 2 mM glutamine for 18 hours at 37°C in 5% CO_2 _in the absence/presence of 100 ng/ml purified endotoxin (lipopolysaccharide (LPS)) derived from *Escherichia coli *O144:H4 (Sigma Co.). After incubation, cell supernatants were collected and kept refrigerated at -70°C until assayed for cytokines. Each day of the experiment, four well controls were applied with monocytes isolated from two healthy volunteers.

The concentrations of TNFα, IL-6 and IL-8 in sera were estimated by an enzyme immunoabsorbent assay (Diaclone, Paris, France). The lowest limits of detection were 0.5 pg/ml for TNFα, 6.25 pg/ml for IL-6 and 62.5 pg/ml for IL-8. The intra-assay and inter-assay variation coefficient of the assays were 4.6 and 5.8% for TNFα, 4.6 and 12.1% for IL-6, and 5.0 and 11.1% for IL-8. Concentrations of TNFα and of IL-6 were also estimated in cell supernatants and expressed as pg/10^4 ^of live cells. The function of monocytes was determined after subtracting the concentrations of TNFα and IL-6 following incubation in the presence of LPS from the concentrations of TNFα and IL-6 following incubation in the absence of LPS.

### Statistical analysis

Results are expressed as medians ± 95% confidence intervals or medians ± standard error. Comparisons were performed by the Mann-Whitney *U *test after correction with the Bonferroni test. All patients were divided into two groups according to monocyte apoptosis on the first day: ≤50% and >50%. The selection of 50% as a cutoff point was based on designing receiver-operating characteristic curves for survival with cutoff values of 40%, 50% and 60%. The greatest area under the curve was generated with a cutoff value of 50%, which was applied in the study.

In order for apoptosis of monocytes to constitute a separate pathophysiological mechanism in sepsis, it should probably be correlated to survival. For that purpose, the survival of each group was estimated by Kaplan-Meyer analysis; comparisons were performed by the log-rank test. Statistical correlations were assayed after assessment of the nonparametric Spearman coefficient (*r*_s_). Monocyte apoptosis between patients who died or who eventually survived was compared by Fischer's exact test. *P *< 0.05 was considered statistically significant.

## Results

Clinical characteristics of patients enrolled in the study are presented in Table 1. Three patients were chronic abusers of alcohol and 34 patients were chronic cigarette smokers, corresponding to 35.71% and 42.42% of patients with apoptosis of monocytes ≤50% and >50% on the first day (*P *= not significant). VAP was not microbiologically documented in 10 patients with sepsis, in eight patients with severe sepsis and in 14 patients with septic shock. Kaplan-Meier estimates of survival over 28 days of follow-up of the entire patient population in correlation to apoptosis of monocytes on the first day showed that patients with monocyte apoptosis on the first day >50% survived longer than patients with monocyte apoptosis ≤50% (*P *= 0.049). Death supervened in 28 patients with apoptosis ≤50% (mortality 49.12%) and in five patients with apoptosis >50% (mortality 15.15%, *P *< 0.0001).

**Figure 2:**
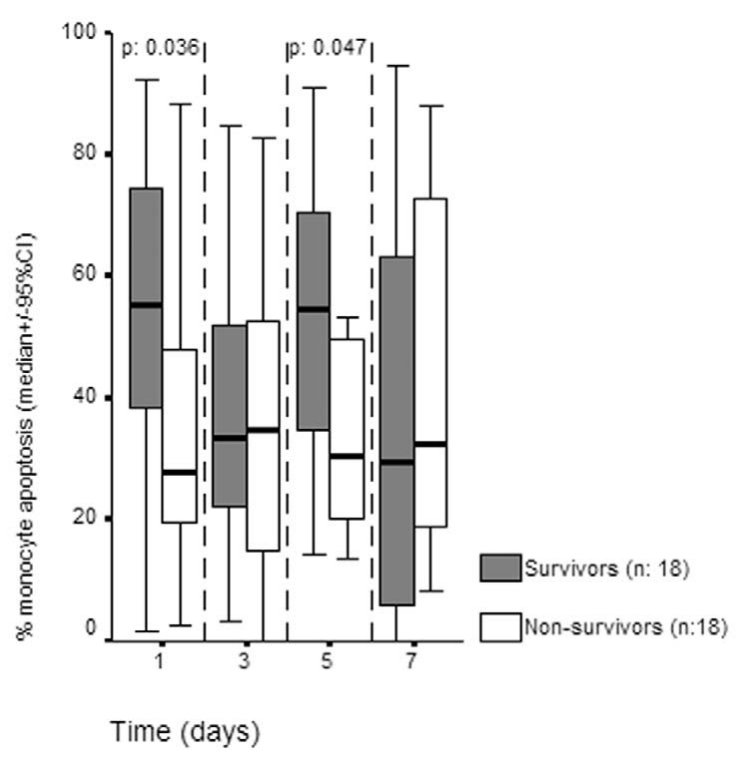
Apoptosis of monocytes of 36 patients with septic shock in relation to final outcome. Circles denote outliers and asterisks extremes.

The median ± standard error monocyte apoptosis on the first day of survivors with sepsis was 37.74 ± 5.81%, and that of nonsurvivors was 11.71 ± 8.24% (*P *= not significant). The respective values for patients with severe sepsis were 44.43 ± 7.18% and 38.11 ± 9.63% (*P *= not significant), and those for patients with septic shock were 55.43 ± 7.11% and 27.61 ± 6.79% (*P *= 0.036). Apoptosis of monocytes of the four healthy volunteers ranged between 5.0% and 9.2%. Changes of monocyte apoptosis over time in relation to the final outcome in patients with septic shock are shown in Figure 2.

**Figure 3:**
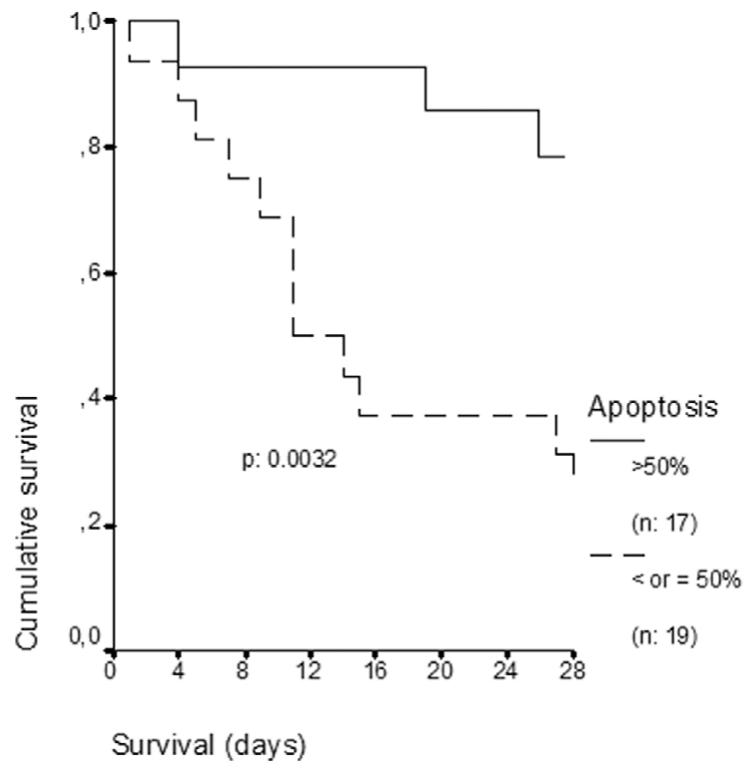
Comparative survival of 36 patients with septic shock in relation to monocyte apoptosis of the first day (≤50% or >50%).

Six patients with sepsis and monocyte apoptosis ≤50% on the first day died (mortality 15%), but no patient with sepsis and monocyte apoptosis >50% died (mortality 0%, *P *= not significant between groups). Seven patients with severe sepsis and monocyte apoptosis ≤50% on the first day died (mortality 38.9%), while two patients with severe sepsis and monocyte apoptosis >50% died (mortality 22.2%, *P *= not significant between groups). Fifteen patients with septic shock and monocyte apoptosis ≤50% on the first day died (mortality 78.9%), and three patients with septic shock and monocyte apoptosis >50% died (mortality 17.64%, *P *= 0.0001 between groups). Kaplan-Meier estimates of survival over 28 days of follow-up in correlation to apoptosis on the first day for 36 patients with septic shock are shown in Figure 3; those with monocyte apoptosis >50% survived longer than those with apoptosis ≤50% (*P *= 0.0032 between groups).

Median apoptosis of monocytes on the first day of patients with bacteraemia was 55.96%, and that of patients without bacteraemia was 36.06% (*P *= 0.020). Moreover, bacteraemia was found in four patients with monocyte apoptosis ≤50% on the first day (7.02%) and in eight patients with apoptosis >50% (24.2%, *P *= 0.027 between groups of apoptosis).

**Figure 4:**
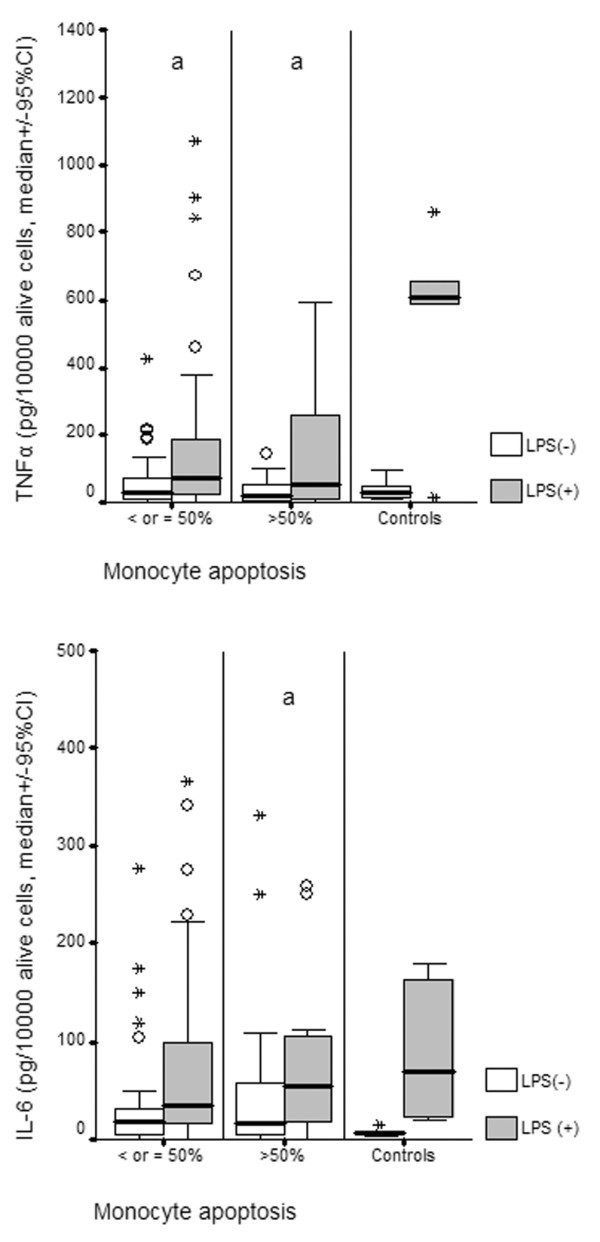
Ex vivo production of TNFα and IL-6 by first day monocytes in relation to apoptosis, without (-) / with (+) triggering by LPS. Circles denote outliers and asterisks extremes. a: response lower than controls

Production of TNFα and IL-6 of blood monocytes of patients isolated from the entire study population on the first day of symptoms in relation to their apoptosis are shown in Figure 4. Positive correlation was found between production of TNFα after triggering with LPS and monocyte apoptosis on the first day (*r*_s _= +0.236, *P *= 0.032).

**Figure 5:**
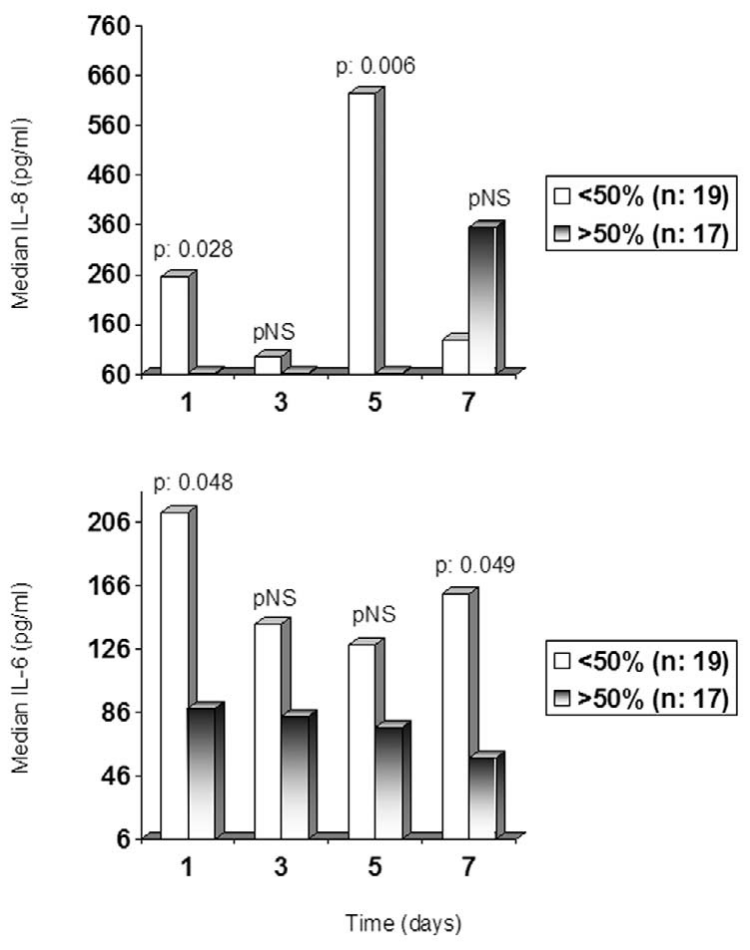
Comparative concentrations of IL-6 and IL-8 over follow-up of patients with septic shock in relation to monocyte apoptosis of the first day

Changes of TNFα, IL-6 and IL-8 of patients' sera in relation to monocyte apoptosis on the first day are presented in Table 2. Positive correlation was found between monocyte apoptosis and concentrations of TNFα on days 3 and 5 (*r*_s _= +0.257, *P *= 0.033 and *r*_s _= +0.257, *P *= 0.044, respectively). Comparative concentrations of IL-6 and IL-8 among patients with septic shock and monocyte apoptosis <50% and ≥50% are shown in Figure 5.

**Figure 6:**
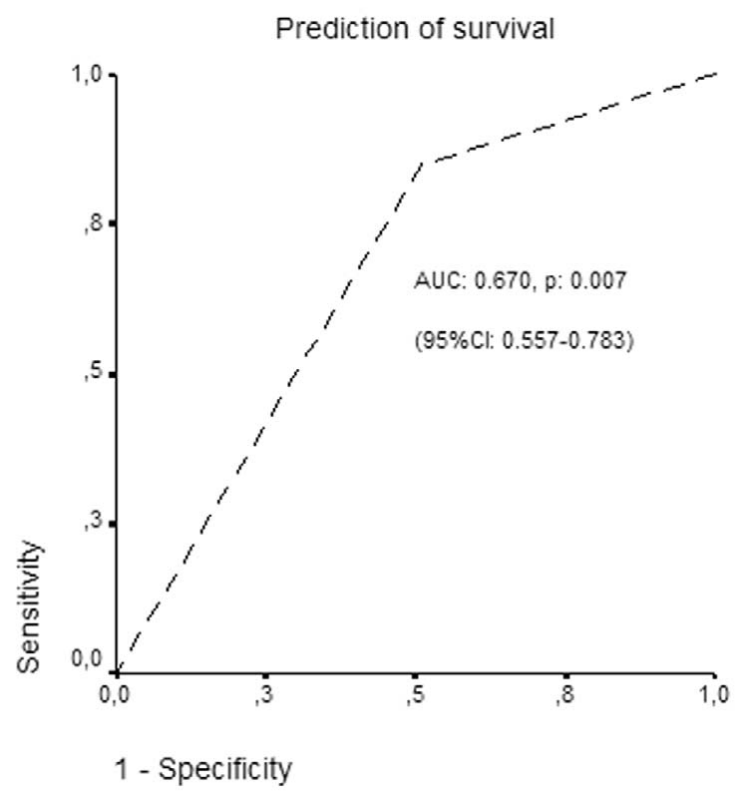
ROC curves of ≥50% apoptosis of monocytes of the first day for survival and for diagnosis of septic shock.

Receiver-operating characteristic curves of apoptosis of monocytes ≤50% for the prediction of final survival are shown in Figure 6.

## Discussion

The present study investigated the existence of apoptosis in blood monocytes over the evolution of sepsis and its correlation to the final outcome of the host. The study design succeeded to eliminate, as much as possible, confounding factors produced when septic patients with various types of infections of probable polymicrobial origin are considered together. Microbiological findings pointed towards a predominance of Gram-negative bacteria as responsible pathogens and towards a monomicrobial cause of VAP (Table 1).

It was proved that apoptosis is a phenomenon taking place in blood monocytes upon presentation of the clinical signs of sepsis (Figure 1). Moreover, it was shown for the first time in the literature that the probability of survival of the septic patient was in parallel to apoptosis on the first day. The latter finding was particularly pronounced in the event of septic shock. Monocyte apoptosis was significantly higher for patients with septic shock who survived compared with those patients with septic shock who died (Figure 2), a phenomenon accompanied by greater mortality among patients with septic shock and apoptosis ≤50% compared with patients with septic shock and apoptosis >50% (Figure 3). In the latter subgroup of patients, differences in apoptosis were still present on day five of follow-up (Figure 2). Moreover, serum levels of proinflammatory cytokines of patients with monocyte apoptosis ≤50% and septic shock on the first day were higher than those of patients with apoptosis >50% and septic shock (Table 2 and Figure 5). These findings are fully compatible with the current theory of the pathogenesis of sepsis elaborating a connection between the production of proinflammatory cytokines by monocytes and the intensity of the inflammatory process [3]. They also lead to the assumption that increased apoptosis of monocytes in the enrolled study population played a protective role for the hosts.

Monocytes isolated on the first day from the entire study population were able to produce proinflammatory cytokines (Figure 4). Due to the initiation of the apoptotic cascade, their functional capacity was decreased compared with monocytes of healthy volunteers. Monocytes with apoptosis ≤50%, however, produced IL-6 at levels similar to monocytes of healthy volunteers. The latter finding is fully compatible with the clinical observation that early monocyte apoptosis offers the host the advantage of prolonged survival because monocytes become less potent to secrete proinflammatory cytokines. The importance of apoptosis of monocytes is further aggravated by the statistical significance of apoptosis greater than 50% for the prediction of survival of the septic patient (Figure 6).

It is not easy to provide one specific mechanism explaining the early triggering of apoptosis in a certain percentage of blood monocytes that would confer protection in the enrolled population of septic patients. Results revealed that bacteraemia was accompanied by elevated apoptosis. This is inconsistent with the finding that elevated monocyte apoptosis favours prolonged survival whereas bacteraemia is connected to poor outcome. Although the total number of patients with bacteraemia is limited, it might be hypothesized that in view of the high risk for death introduced by bacteraemia, monocyte apoptosis is triggered for protection of the host. Moreover, it might be proposed that shed bacterial products act as inducers of apoptosis. It was found that production of TNFα by LPS was correlated to monocyte apoptosis. It might thus be hypothesized that TNFα secreted by monocytes acted by an autocrine mode eliciting apoptosis.

Knowledge of apoptosis of cells of the immune system in sepsis is derived mainly from animal studies. Initiation of apoptosis in the field of a septic process has been demonstrated in tissue macrophages of the lung of animals [14]. Studies in humans have shown considerable depletion of the spleen from B lymphocytes and T lymphocytes at autopsy postmortem [15,16]. Two studies described apoptosis in lymphocytes [17] and monocytes [2] of septic patients. The former correlated increased apoptosis of patients with septic shock with poor outcome [17]. The latter study [2] described reduced membrane potential, which was a finding consistent with initiation of the apoptotic pathway, in blood monocytes of 18 patients with severe sepsis. No connection was found with survival. The present study is the only in the literature presenting the consecutive time evolution of the apoptotic potential in blood cells of the innate immune system in a considerable number of patients, and its correlation to progression to death particularly in the event of septic shock.

The following limitations of the present study should be considered. Analysed monocytes considered necrotic (that is to say, propidium iodine-positive) might be either primary necrotic or secondary necrotic due to apoptosis. All these cells were excluded from the analysis. The findings should also be verified in a larger study population. Finally, any inadequate initial antimicrobial treatment might have had some effect.

## Conclusion

The presented results revealed a connection between early apoptosis of monocytes upon presentation of clinical signs of sepsis and favourable outcome. The presented findings are of particular importance for the patient with septic shock, where they might constitute a mechanism of pathogenesis.

## Key messages

• 	Early apoptosis of blood monocytes at a level greater than 50% is accompanied by prolonged survival of the septic host, a phenomenon pronounced in patients with septic shock.

• 	Apoptotic monocytes to a degree greater than 50% are less potent for the release of proinflammatory cytokines

## Abbreviations

IL = interleukin; LPS = lipopolysaccharide; PBS = phosphate-buffered saline; pO_2_/FiO_2 _= partial oxygen pressure/fraction of inspired oxygen; TBS= tracheobronchial secretions; TNFα = tumour necrosis factor alpha; VAP = ventilator-associated pneumonia.

## Competing interests

The authors declare that they have no competing interests.

## Authors' contributions

EJGM participated in the study design, coordinated the laboratory procedures, analysed the data and wrote the manuscript. CR participated in the study design and in the follow-up of patients. DP, MR and VK participated in the follow-up of patients and in the laboratory procedures. VM, DZ, SO and AK participated in the follow-up of patients. AA, CR and HG drafted the manuscript.

## Figures and Tables

**Table 1:** Clinical characteristics of patients with ventilator-associated pneumonia enrolled in the study categorized according to the ACCP/SCCM classification.

Characteristic	Sepsis	Severe sepsis	Septic shock
Number of patients	27	27	36
Age (years, mean ± SD)	52.6 ± 20.8	67.7 ± 13.6	58.0 ± 18.1
Male/female	20/7	18/9	27/9
APACHE II score (mean ± SD)	15.63 ± 6.06	17.88 ± 4.19	19.40 ± 7.03*
Sequential Organ Failure Assessment score (mean ± SD)	5.78 ± 2.82	7.21 ± 2.83	9.54 ± 4.10^†^
White blood cells (/μl, mean ± SD)	12.132,6 ± 5.060,7	13.850,0 ± 7.026,9	14.206,0 ± 8.683,0
Underlying conditions [*n *(%)]			
Multiple trauma	8 (29.62)	1 (3.70)	9 (25.00)
Brain haemorrhage	6 (22.22)	7 (25.92)	3 (8.33)
Respiratory failure due to chronic obstructive pulmonary diseases	8 (29.62)	8 (29.62)	11 (30.55)
Acute abdomen	1 (3.70)	2 (7.41)	4 (11.11)
Celiac aorta aneurysm replacement	-	2 (7.41)	3 (8.33)
Others	4 (14.81)	7 (25.92)	6 (16.66)
Predisposing factors [*n *(%)]			
Diabetes mellitus type 2	4 (14.81)	4 (14.81)	6 (16.66)
Coronary heart disease	3 (11.11)	8 (29.62)	4 (11.11)
Hypertension	2 (7.41)	6 (22.22)	4 (11.11)
Others	6 (22.22)	-	8 (22.22)
Pathogens [*n *(%)]			
*Acinetobacter baumannii*	10 (37.03)	11 (40.74)	16 (44.44)
*Pseudomonas aeruginosa*	5 (18.51)	7 (25.92)	4 (11.11)
Others	2 (7.41)	3 (11.11)	2 (5.55)
Bacteraemia [*n *(%)]	4 (14.81)	3 (11.11)	5 (13.88)
Case fatality [*n *(%)]	6 (22.2)	9 (33.3)	18 (50.0)

APACHE, Acute Physiology and Chronic Health Evaluation; SD, standard deviation. *P = 0.017 compared with patients with sepsis. ^†^*P *= <0.0001 compared with patients with sepsis and *P *= 0.011 compared with patients with severe sepsis.

**Table 2:** Concentrations of tumour necrosis factor-alpha (TNFα), interleukin(IL)-6 and IL-8 in relation to monocyte apoptosis (≤50% or >50%) of the first day

Day	Sepsis		Severe sepsis		Septic shock	
	
	≤50% (*n *= 20)	>50% (*n *= 7)	≤50% (*n *= 18)	>50% (*n *= 9)	≤50% (*n *= 19)	>50% (*n *= 17)
Tumour necrosis factor alpha						
1	4.97 ± 1.91	4.95 ± 0.20	6.84 ± 0.97	7.33 ± 28.20	6.98 ± 11.21	6.85 ± 3.29
3	7.28 ± 4.39	5.23 ± 19.50	5.70 ± 37.30	5.16 ± 1.48	5.15 ± 14.45	9.98 ± 1.74
5	5.33 ± 0.86	5.08 ± 5.03	6.37 ± 10.92	7.39 ± 2.26	10.32 ± 30.03	9.36 ± 7.52
7	6.22 ± 4.37	6.58 ± 0.63	4.83 ± 3.23	5.92 ± 2.22	12.44 ± 18.41	6.12 ± 3.20*
IL-6						
1	95.4 ± 24.2	123.1 ± 61.1	102.9 ± 20.3	69.4 ± 26.4	212.3 ± 30.7	88.3 ± 25.3*
3	80.1 ± 21.2	63.7 ± 27.7	96.6 ± 26.1	114.6 ± 31.8	141.2 ± 25.7	83.2 ± 22.1
5	89.4 ± 25.4	112.2 ± 50.1	96.5 ± 23.1	98.7 ± 38.3	128.2 ± 36.0	76.5 ± 23.4
7	61.3 ± 21.3	41.4 ± 49.5	107.2 ± 24.7	99.9 ± 27.9	160.4 ± 30.2	57.4 ± 23.6*
IL-8						
1	181.5 ± 152.9	403.6 ± 315.1	62.5 ± 33.3	62.5 ± 143.2	255.4 ± 126.5	62.5 ± 72.7*
3	152.4 ± 145.2	62.5 ± 91.2	62.5 ± 31.8	62.5 ± 230.9	95.3 ± 140.8	62.5 ± 119.7
5	62.5 ± 54.7	423.5 ± 103.2	62.5 ± 70.0	314.6 ± 210.2	623.8 ± 206.6	62.5 ± 123.5*
7	99.9 ± 114.4	62.5 ± 80.9	62.5 ± 88.9	108.1 ± 281.6	128.1 ± 281.6	355.2 ± 94.4

Data presented as the median ± standard error (pg/ml). *P < 0.05 compared with patients of the same group with apoptosis ≤50%.
